# Gene network analysis of poplar root transcriptome in response to drought stress identifies a *PtaJAZ3PtaRAP2*.*6*-centered hierarchical network

**DOI:** 10.1371/journal.pone.0208560

**Published:** 2018-12-12

**Authors:** Madhumita Dash, Yordan S. Yordanov, Tatyana Georgieva, Hairong Wei, Victor Busov

**Affiliations:** Forest Resources and Environmental Science, Michigan Technological University, Houghton, MI, United States of America; National Taiwan University, TAIWAN

## Abstract

Using time-series transcriptomic data from poplar roots undergoing polyethylene glycol (PEG)-induced drought stress, we built a genetic network model of the involved putative molecular responses. We found that the network resembled a hierarchical structure. The highest hierarchical level in this structure is occupied by 9 genes, which we called superhubs because they were primarily connected to 18 hub genes, which are then connected to 2,934 terminal genes. We were only able to regenerate transgenic plants overexpressing two of the superhubs, suggesting that the majority of the superhubs might interfere with the regeneration process and did not allow recovery of transgenic plants. The two superhubs encode proteins with closest homology to *JAZ3* and *RAP2*.*6* genes of *Arabidopsis* and were consequently named *PtaJAZ3* and *PtaRAP2*.*6*. *PtaJAZ3* and *PtaRAP2*.*6* overexpressing transgenic lines showed a significant increase in both root elongation and lateral root proliferation and these responses were specific for the drought stress conditions and were highly correlated with the levels of overexpression of the transgenes. Several lines of evidence suggest of regulatory interactions between the two superhubs. Both superhubs were significantly induced by methyl jasmonate (MeJA). Because jasmonate signaling involves ubiquitin-mediated proteasome degradation, treatment with proteasome inhibitor abolished the MeJA induction for both genes. *PtaRAP2*.*6* was upregulated in *PtaJAZ3* transgenics but *PtaJAZ3* expression was not affected in the *PtaRAP2*.*6* overexpressors. The discovery of the two genes and further future insights into the associated mechanisms can lead to improved understanding and novel approaches to regulate root architecture in relation to drought stress.

## Introduction

Drought is a major abiotic stress that affects plant development and productivity and is predicted to intensify in the coming decades [1–3]. Therefore, breeding and/or deploying drought resistance technologies in agricultural, forestry and bioenergy industries requires an understanding on the underlying molecular regulatory mechanisms controlling plants resistance to drought stress. This is particularly important for bioenergy crops like poplar, which is usually grown on marginal lands to avoid competition with arable lands [4, 5].

Molecular and physiological studies have identified multiple plant responses to drought stress occurring in different organs and tissues [6–9]. Changes in root growth and architecture play a major role in plant adaptation to drought stress [10–14]. As a result, changes and adjustment in root architecture to dehydration stress have become major focal areas of research for improving drought resistance in crop plants [15–19]. Molecular genetics approaches, to date, have identified only a few genes that modulate or augment drought tolerance via changes in root architecture [20–24]. Knowledge regarding the complex regulatory networks associated with roots’ response to drought stress is still limited and this precludes the use of more effective approaches to develop drought resistant varieties through root system architecture engineering.

Several transcriptomic analyses, both in poplar and other plant species, have successfully identified genome-wide transcriptional changes in roots [25–28] and other tissues [29–32] in response to drought stress. In most of these studies, drought responses were studied across different genotypes [25, 31, 33, 34] or at different levels of drought stress [25, 28]. However, most of these studies employ a single time sampling design, which does not allow understanding the temporal patterns in developing the response. Indeed, it has been shown that studying drought stress for a single time point is insufficient to fully understand the transcriptional network associated with drought response [32].

In contrast, time-course experimental design provides a dynamic and progressive view of molecular events across multiple time points during the whole response process. Time-series transcriptomic data is thus better-suited for inferring the underlying genetic regulatory networks (GRNs) because enables mathematical modeling that draws inferences about the underlying gene interactions and hierarchical regulatory networks [35–37]. These approaches, however, have been rarely and only recently used in understanding root development [38–40] and have not been used to date for understanding the GRNs involved in root response to drought stress. We have recently shown the power of this approach by using time-series microarray data of poplar roots undergoing response to low nitrogen condition [38, 40]. The modeling of the GRNs enabled identification of a new regulatory module. Transgenic manipulation of genes from this module had a profound effect on root growth and performance under low nitrogen.

In this study, we used a time-course transcriptome approach coupled with gene network inference and network analysis to identify regulatory mechanisms modifying root architecture in response to PEG-induced drought stress in poplar.

## Materials and methods

### Plant material and PEG treatments

All experiments were performed in the *Populus tremula x Populus alba* INRA 717-IB4 genotype (wildtype or WT-717) that were maintained *in vitro* on ½ MS media with 20 g/l sucrose (Caisson, https://www.caissonlabs.com/), 0.1 mg/l Indole butyric acid (IBA, Sigma-Aldrich, https://www.sigmaaldrich.com/), vitamins solidified with 2.5 g/l Gelrite (Sigma) and 4 g/l Phytablend agar (Caisson).

For the time-course experiment of PEG treatment was performed in liquid media. The top three internodes (leaves removed) of *in vitro* propagated plants were placed on filter paper bridges in glass tubes filled with 15 ml of liquid ½ MS media (without IBA) and were allowed to develop root system for three weeks. Plants with uniform root growth were then further transferred to control (½ MS media) or PEG liquid media containing 5% polyethylene glycol (PEG) 6000 (Sigma). Roots were sampled at 0, 6, 24, 48, 96 and 504h after transfer to control and PEG liquid media and stored at -80°C until further processed.

The response of all the transgenic lines to PEG treatment was also performed following the screening procedure described above but with slight modification as described previously [24]. Briefly, single node of *in vitro* growing poplar plants were grown on solid medium with high IBA concentration (2 mg/l) for 7 days at 22 ^o^C in dark. After high IBA treatment explants were directly transferred to control and PEG liquid media and grown for 40 days. Roots were scanned and analyzed using ImageJ software. After scanning the roots were stored at -80 ^o^C until further processed.

### Microarray analysis and quantitative RT-PCR

Two independent biological replicates were used for each treatment. Total RNA was extracted from poplar root tissues using a modified RNeasy Mini Kit (Qiagen) as described previously [41]. RNA quality was assessed using Agilent Bionalayzer and 0.2 μg of total RNA was used for further microarray analysis. Microarray analysis were performed using the Affymetrix Poplar GeneChip (Affymetrix) as described previously [42]. Microarray data from this study has been deposited into the Gene Expression Omnibus (GEO) database at NIH (http://www.ncbi.nlm.nih.gov/geo/) with accession number GSE116922.

Data normalization was performed using Robust Multichip Average (RMA) analysis [43]. Differentially expressed genes (DEGs) were identified using Rank Product [44] analysis followed by multiple corrections using Benjamini Hochberg False Discovery Rate [45]. Gene annotation of Poplar Affymetrix Chips was accomplished by blasting the target sequences of probe sets to *Populus trichocarpa* V3.0 (phytozome.org) transcripts. The target sequences were the poplar cDNA and EST sequences that were used to design probe sets and were provided by Affymetrix. The homologous counterparts of *Populus trichocarpa* V3.0 transcripts in *Arabidopsis thaliana* were provided in *Populus trichocarpa* V3.0annotation information file downloaded from phytozome.net, via which Affymetrix probes are mapped to *A*. *thaliana* genes.

Gene expression was analyzed by quantitative RT-PCR using the StepOnePlus Real Time System (Applied Biosystems) as described previously [38]. Gene-specific primers used in this study are indicated in S1 Table. Primer efficiency was determined using cDNA dilution series and the efficiencies ranged from 1.83 to 1.96. Gene expression was normalized using *Ubiquitin* as a reference gene.

### Gene network construction and gene ontology analysis

The global gene network was constructed as described previously [38, 42]. Briefly, the expression profiles of the identified 5,607 DEGs and a list of differentially expressed transcription factors (TF) were used as input for constructing gene regulatory networks using the Algorithm for the Reconstruction of Accurate Cellular Networks (ARACNE) [46]. The gene association network built with ARACNE was then further searched to identify TFs that are connected to three to fifteen hub genes using an in-house Perl script.

Gene ontology analysis was performed using agriGo’s SEA (Singular Enrichment Analysis) tool available at (http://bioinfo.cau.edu.cn/agriGO/). SEA was coupled with available background data of Populus Affymetrix Genome Array and GO terms with a corrected p-value or Hochberg (FDR) value < 0.001 were considered as significantly enriched. In order to visualize the temporal changes in the functional categories, all the DEGs were subjected to over/under-representation analysis using PageMan tool [47]. Log_2_ fold change of all the up-regulated and down-regulated DEGs was used as input for the PageMan analysis. Wilcoxon rank sum tests with Benjamini and Hochberg correction was used to detect over- or under-represented functional categories among different DEGs. The adjusted p-values produced were then transformed into their respective z-values where a z-score of 0 means p-value > 0.05. The resulting values were then false color coded using a color scale of -4 to 4 and higher color intensity represents lower p-value.

### Binary vector generation and plant transformation

Generation of overexpression constructs were performed using Gateway technology as previously described [48]. Gene specific primers with attached attB sequence were used to amplify open reading frames of the superhubs (S2 Table). Entry clones were constructed using pDONR221 vector which were then further cloned into binary vectors pK7m24GW3 (overexpression) using BP and LR clonases (Invitrogen, Life Technologies) respectively. *Agrobacterium*-mediated transformation was performed as described previously [49]. All overexpression transgenic lines were verified using PCR followed by TaqI digestion.

### Jasmonate and proteasome inhibitor treatments

Methyl Jasmonate (MeJA) was used for the jasmonate treatments. MeJA stock solution was prepared using 95% aqueous MeJA solution (Sigma) and dimethylsulfoxide (DMSO) (Acros, http://www.acros.com/) as a solvent. After the plants have grown in the liquid media for 40 days, 50 μM of MeJA was added to the control and PEG liquid media. Similarly, proteasome inhibitor MG132 (Reagents Direct, http://www.reagentsdirect.com/) was used to treat WT-717 and transgenic plants growing in control and PEG liquid media (see above). Proteasome inhibition was performed by adding 80 μM of MG-132 and 80 μM of MeJA to the liquid media. The plants were grown in the MeJA, MeJA + MG-132 and DMSO-containing media for an additional 24 h. Roots were then flash frozen in liquid nitrogen and stored at -80°C for further analyses.

## Results

### Significant changes in root morphology and transcriptome in response to drought stress

We have previously shown that lateral root (LR) growth is severely inhibited in response to drought stress elicited by PEG treatment [24]. In order to understand the mechanisms underpinning this response and identify genes that may overcome LR inhibition we studied the temporal transcriptomic changes in poplar roots undergoing PEG-induced drought stress (see experimental procedures). Over the whole experimental period that comprises of six time points, a total of 5,607 genes were identified to be differentially expressed in response to the PEG treatment (S1 Data). The number of differentially expressed genes (DEGs) peaked as early as 6h post treatment and gradually decreased over time (Table 1). The changes in expression were successfully validated for 12 genes using qRT-PCR (S1 Fig).

**Table 1:** Temporal changes in the number of differentially expressed genes (DEGs) in poplar roots in response to PEG-induced drought stress.

Time	6h	24h	48h	96h	504h
Total DEG	2499	1835	1633	784	482
Up-regulated	963	671	746	273	126
Down-regulated	1536	1164	887	511	356

Complete list of the genes and their expression values are provided in Data S1.

### Functional categories associated with drought stress

**Fig 1:**
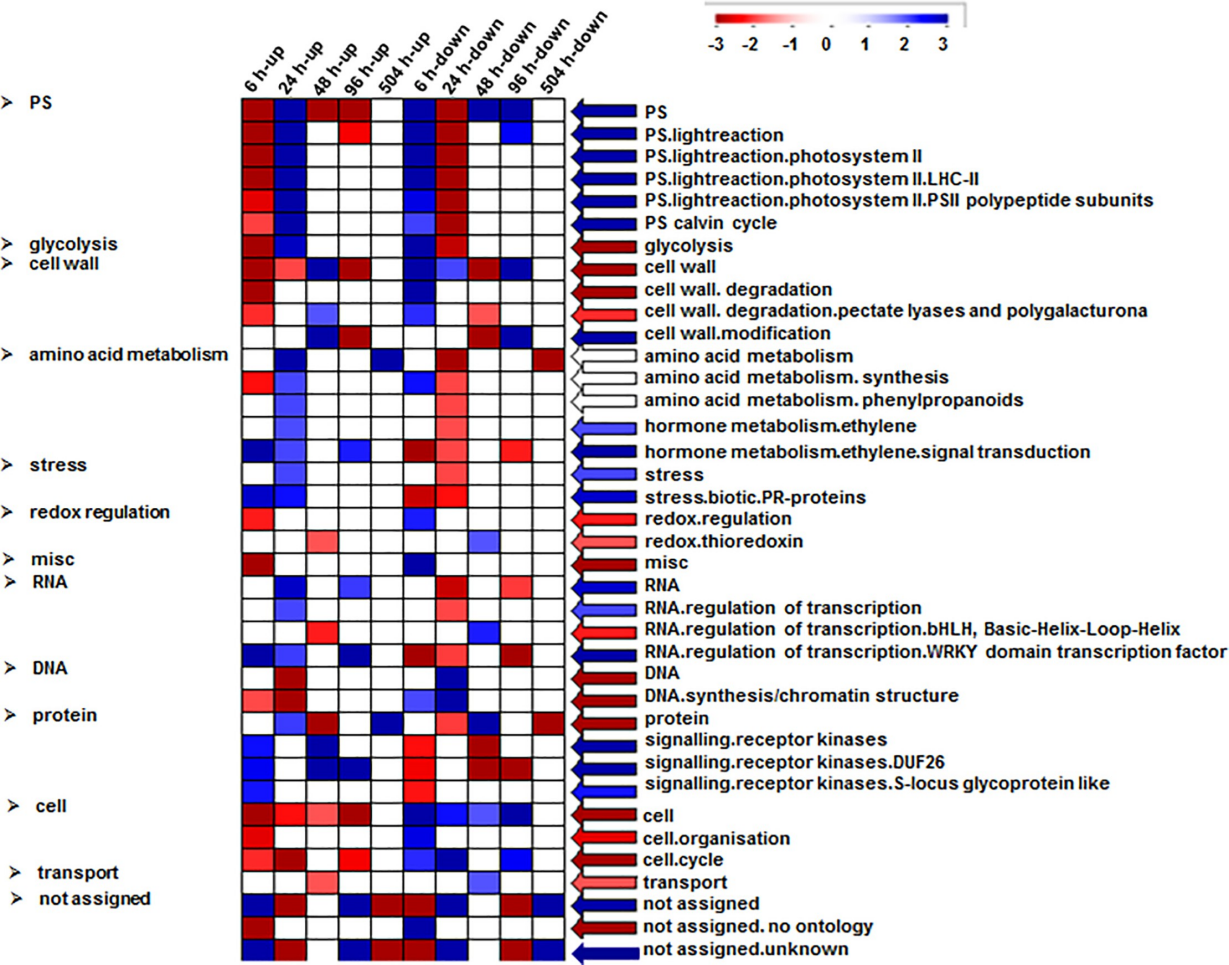
PageMan display of significantly represented functional categories across different time points in response to PEG treatment. Differentially expressed genes (DEGs) for various time points displaying significant up- and down-regulation were used for the analysis (see materials and methods). The data were subjected to a Wilcoxon test, and the results are displayed as blue-colored bins (significantly over represented), red-colored bins (significantly under represented) and white-colored bin (not significant). Arrow at right side of heatmap represents average level of significance for all the time points. In the figure, non-significant categories have been collapsed to display only significant functional categories. The main functional categories are displayed in the left whereas specific sub-categories are shown in the right.

Gene enrichment analysis was conducted to identify the biological processes associated with the observed overall and temporally-specific changes. The biological processes that were overrepresented in all the DEGs were those that are associated with metabolic processes and response to stimulus (S2 Data). The molecular functions significantly enriched were associated with binding activity (co-factor binding, metal ion binding, *etc*.), catalytic activity, antioxidant activity and transferase activity. Inspection of the temporal changes in the processes associated with the DEGs showed interesting trends and dynamics (Fig 1). For example, the first two time points showed opposite enrichment patterns for several functional categories, like those associated with photosystem, glycolysis, cell wall modification/degradation and amino acid metabolism (Fig 1). Genes associated with these categories were suppressed at 6h whereas at 24h their expression was increased, suggesting very fast and essentially full reprogramming for these functional categories over a short period of time during the progressive course of the drought stress.

### Transcriptome changes in response to drought stress are hierarchically structured

To identify important genes that govern root architectural changes in response to drought, we performed gene regulatory network (GRN) analysis. We identified a highly organized three-tiered network centered on nine genes, which we called superhubs (S2 Fig). The nine superhubs were connected to 18 hub genes, which were then connected to 2,934 terminal genes (S2 Fig and S3 Data). Thus, this structure encompassed more than 50% of the differentially expressed genes (total number of DEGs was 5,607). Since, approximately half of the network was centered on these nine superhub genes, we hypothesized that they may play major role in orchestrating the responses. To better understand the function of the nine superhubs, we generated constructs targeting overexpression of the nine genes. We transformed these constructs into poplar.

### Overexpression of *PtaJAZ3* and *PtaRAP2*.*6* superhubs increases root growth under drought stress

**Fig 2:**
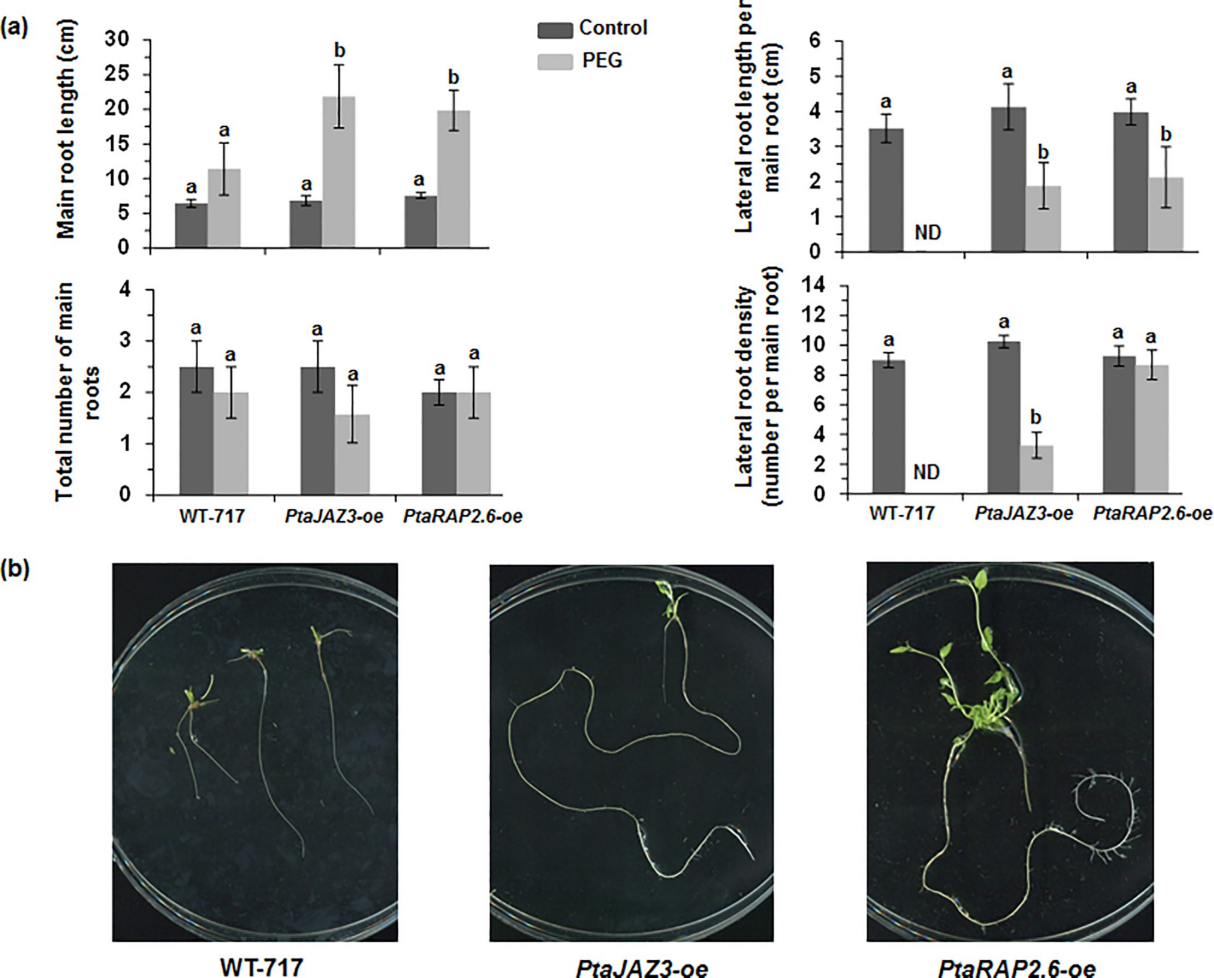
*PtaJAZ3* and *PtaRAP2*.*6* overexpression affects root growth in response to PEG-induced drought stress. (a) Root growth of *PtaJAZ3* and *PtaRAP2*.*6* overexpressing transgenics in comparison with those of WT-717 in both control (darker bars) and PEG (lighter bars) media. Three independent lines (four replicates per line, see S3 Fig) were measured for calculating the values of each transgenic modification that is shown as mean ± standard error of mean (SEM) (*n* = 3). Different letters represent means that are statistically different (*P*< 0.05) as determined by a one-way ANOVA followed by Tukey’s multiple range tests. (b) Representative photos of roots from the three genotypes grown in PEG liquid media for 40 days (see materials and methods).

**Fig 3:**
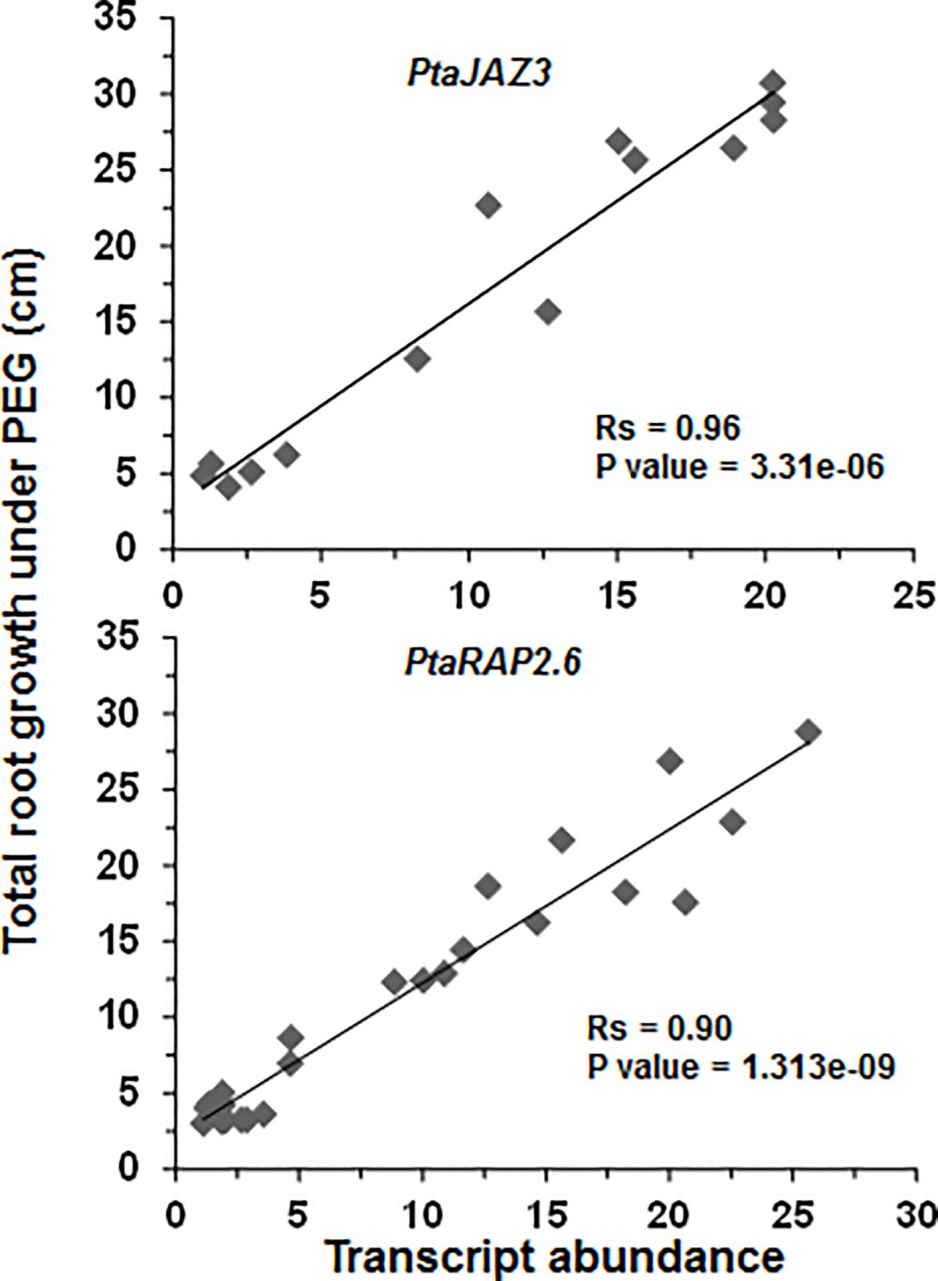
Level of *PtaJAZ3* and *PtaRAP2*.*6* transgenic overexpression is correlated to the increased root growth under PEG. Spearman rank correlation (Rs) between relative transgene expression and total root growth in *PtaJAZ3* and *PtaRAP2*.*6* transgenic lines. For correlation analysis, 14 and 25 independent transgenic lines were used for *PtaJAZ3-oe* and *PtaRAP2*.*6-oe*, respectively. Analysis was performed in plant roots growing in liquid media (see materials and methods for details). Total root growth represents sum of main root length and lateral root length for each plant. Transcript abundance and total root growth was analyzed in 4 replicates per transgenic line.

Majority of the overexpression constructs (7 out of 9) showed very low regeneration ability, indicating that these genes are strong developmental regulators. We were able to generate sufficient transgenic plants (more than 10 independent events) for only two superhubs. These two superhubs encode, (1) a transcriptional repressor with closest homology to *Arabidopsis Jasmonate-Zim-Domain protein 3* (*PtaJAZ3*) and (2) a transcription factor with closest homology to *RAP2*.*6* (*Related to Apetala 2*.*6*) (Fig 2). Overexpression for both superhubgenes was confirmed through qRT-PCR (S3 Fig). We next studied the response of the overexpressing transgenic lines in both control and drought stress conditions, as used for the transcriptome profiling. Under control conditions, both *PtaJAZ3* and *PtaRAP2*.*6* overexpressors did not show any difference of root development when compared to wildtype (Fig 2). However, both *PtaJAZ3* and *PtaRAP2*.*6* overexpressing transgenics displayed increased root growth under PEG conditions (Fig 2) which included increased main root length, lateral root length and lateral root density. The increase in total root growth under PEG conditions was highly quantitatively correlated to the level of overexpression of the two superhubs (Fig 3).

### *PtaJAZ3* and *PtaRAP2*.*6* centered subnetworks encompass similar biological processes

**Fig 4:**
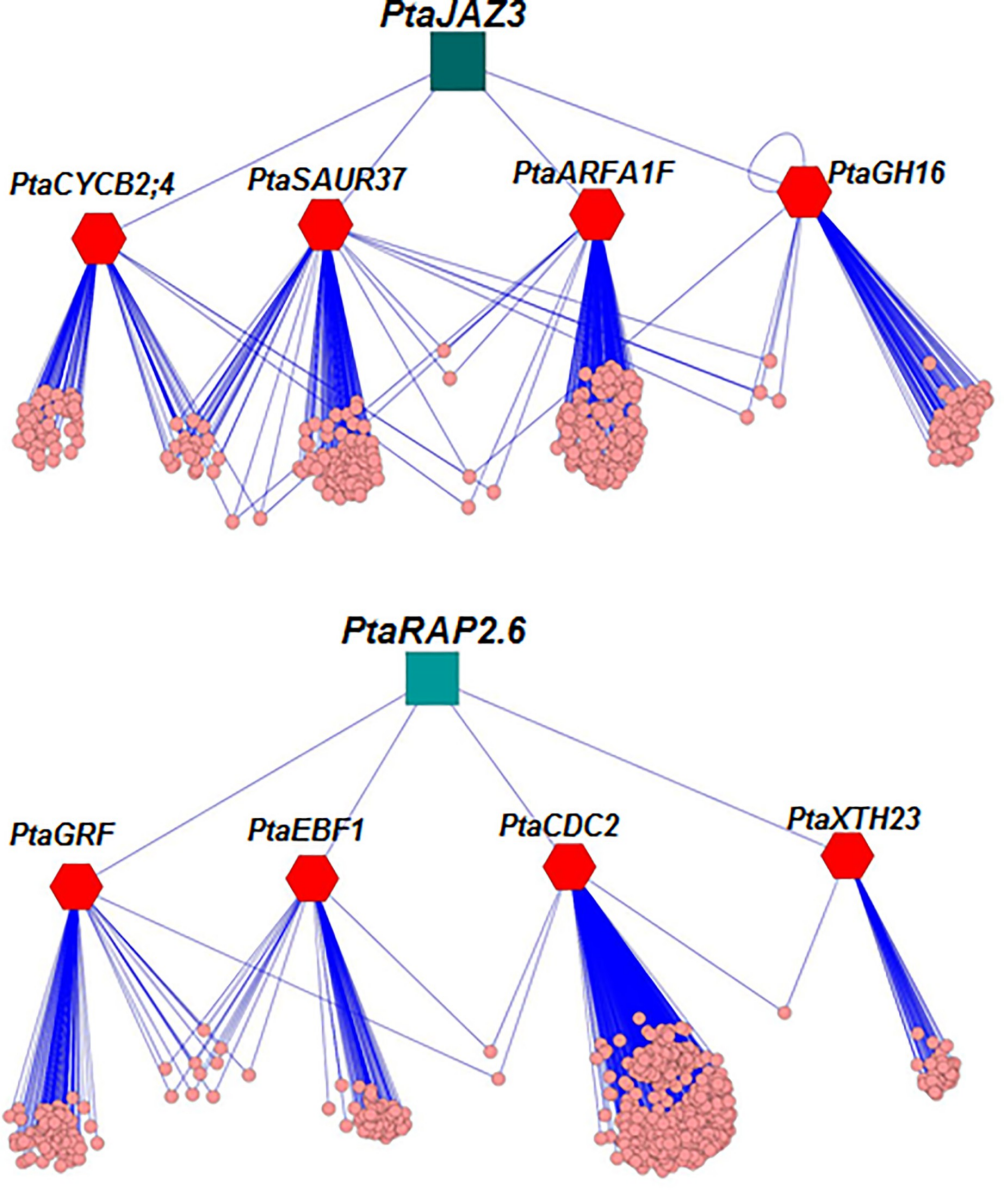
*PtaJAZ3*- and *PtaRAP2*.*6-*centered networks. Both the regulatory networks were generated using Algorithm for the Reconstruction of Accurate Cellular Networks (ARACNE) analysis (see experimental procedures). The two superhubs (squares) were connected to four hub genes (hexagons) which in turn were connected to terminal genes (circles). Abbreviations for the four hubs connected to *PtaJAZ3* are as follows: 1. *PtaCYCB2;4* (Cycline B2;4, Potri.005G251400); 2. *PtaSAUR37* (Small Auxin Upregulated RNA 37, Potri.006G278100); 3.*PtaARFA1F* (ADP-Ribosylation Factor A1F, Potri.008G100000); 4. *PtaGH16* (Glycosyl Hydrolases family 16, Potri.006G071200). Abbreviations for the four hubs connected to *PtaRAP2*.*6* are as follows: 1. *PtaGRF* (Gibberellin-Regulated Family protein, Potri.014G020100); 2. *PtaEBF1* (EIN3-Binding F-box protein1, Potri.006G068500); 3. *PtaCDC2* (Cell Division Control 2, Potri.004G133500); 4. *PtaXTH23* (Xyloglucan Endotransglucosylase/Hydrolase 23, Potri.018G095100). Details of the terminal genes connected to individual hubs are provided in S4 Data and S1 Table.

*PtaJAZ3* and *PtaRAP2*.*6*were connected to four hub genes each that were homologous to genes involved in cell division (*Cyclin B2;4*/*PtaCYCB2;4* and *Cell Division Control 2*/*PtaCDC2*), cell wall metabolism (*XyloglucanEndotransglucosylase Hydrolase 23*/*PtaXTH23*), sugar metabolism (*Glycosyl Hydrolases family 16*/*PtaGH16*), cell signaling (*ADP-Ribosylation Factor A1F*/*PtaARFA1F*) and hormonal signaling (*Small Auxin Upregulated RNA 37*/*PtaSAUR37*, *EIN3-Binding F-box protein1*/*PtaEBF1* and *Gibberellin-Regulated Family protein*/*PtaGRF*) (Fig 4 and S4 Data). Ontology enrichment analysis of the genes involved in these two subnetworks revealed an enrichment of similar biological processes such as regulation of metabolic processes, cell cycle regulation and regulation of gene expression (S3 Table).

### *PtaJAZ3* and *PtaRAP2*.*6* overexpression changes the expression of the hub genes

**Fig 5:**
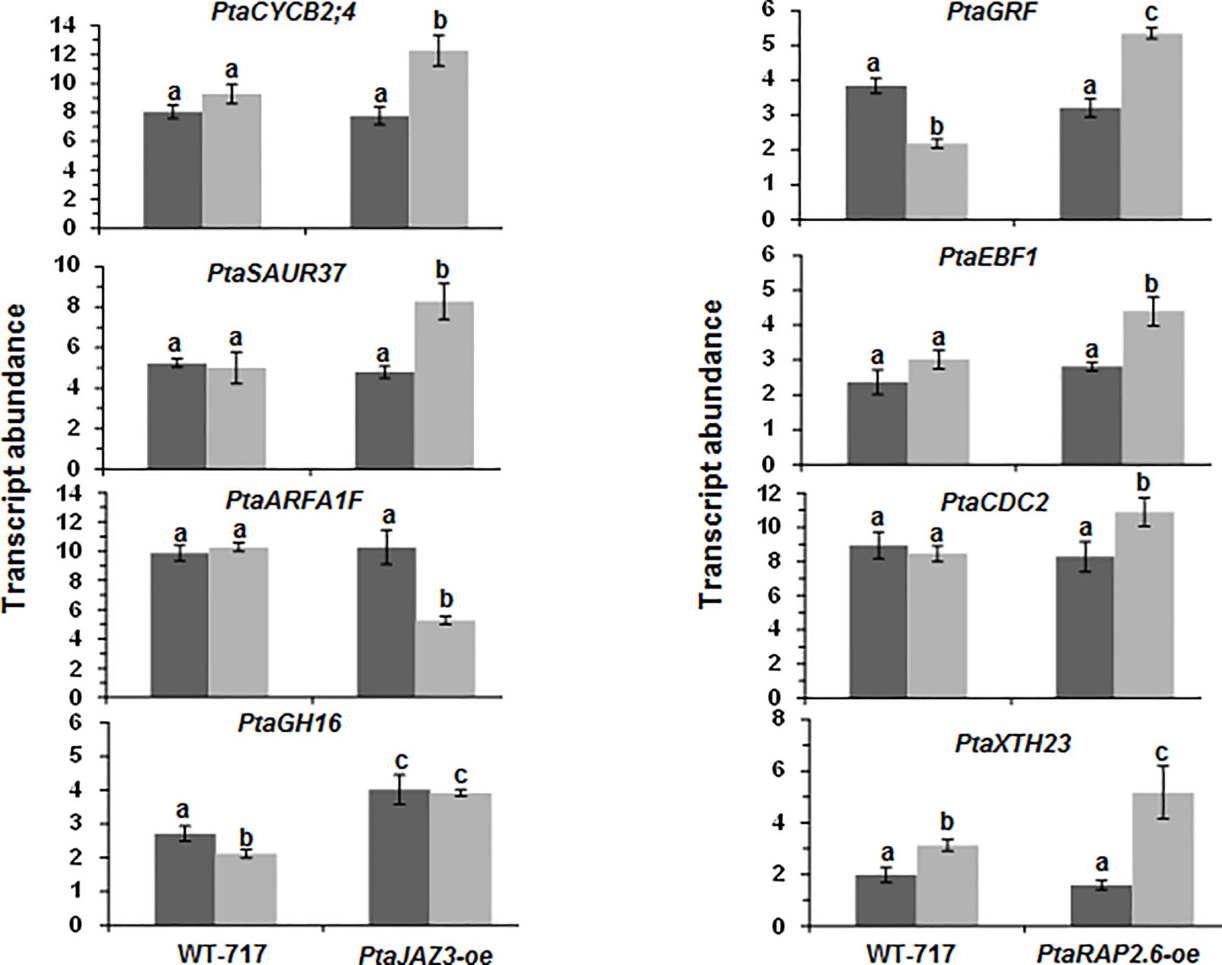
*PtaJAZ3* and *PtaRAP2*.*6* overexpression affects transcript abundance of the respective hub genes in response to PEG treatment. Transcript abundance was analyzed in roots of WT-717, *PtaJAZ3-oe* (left) and *PtaRAP2*.*6-oe* (right) transgenic lines under both control and PEG conditions. Hub genes associated with *PtaJAZ3* and *PtaRAP2*.*6* are shown in the left and right graph panels respectively. Two independent lines (four replicates per line) were used for each transgenic modification. Values show genotypes’ mean ± SEM (*n* = 2). Different letters represent means that are statistically different (*P*< 0.05) as determined by a one-way ANOVA followed by Tukey’s multiple range tests.

To better understand the relation between the superhub and hub genes and to validate that indeed these genes are organized in a hierarchical network, we studied the expression of the hub genes in the superhub overexpression transgenics. Majority of the hubs (7 out of 8) displayed differential expression in the transgenics and this, as with the phenotypic changes of the superhub transgenics, was only observed under PEG conditions (Fig 5). *PtaGH16*, one of the hubs of *PtaJAZ3*, showed differential expression in *PtaJAZ3-oe* under both control as well as PEG conditions.

### *PtaJAZ3* and *PtaRAP2*.*6*showed tissue-specific expression

To better understand the native functions of the two superhub genes, we characterized their tissue-specific expression under control condition. Both *PtaRAP2*.*6*and *PtaJAZ3*were highly expressed in roots compared to other tissues that included shoot apex, leaves and stems (S4 Fig), though the expression level of *PtaJAZ3* in leaves was only slightly lower than that of *PtaJAZ3* in roots. These results implicate that both genes were predominantly expressed in roots.

### *PtaJAZ3* and *PtaRAP2*.*6* expression was affected by drought stress and methyl jasmonate

**Fig 6:**
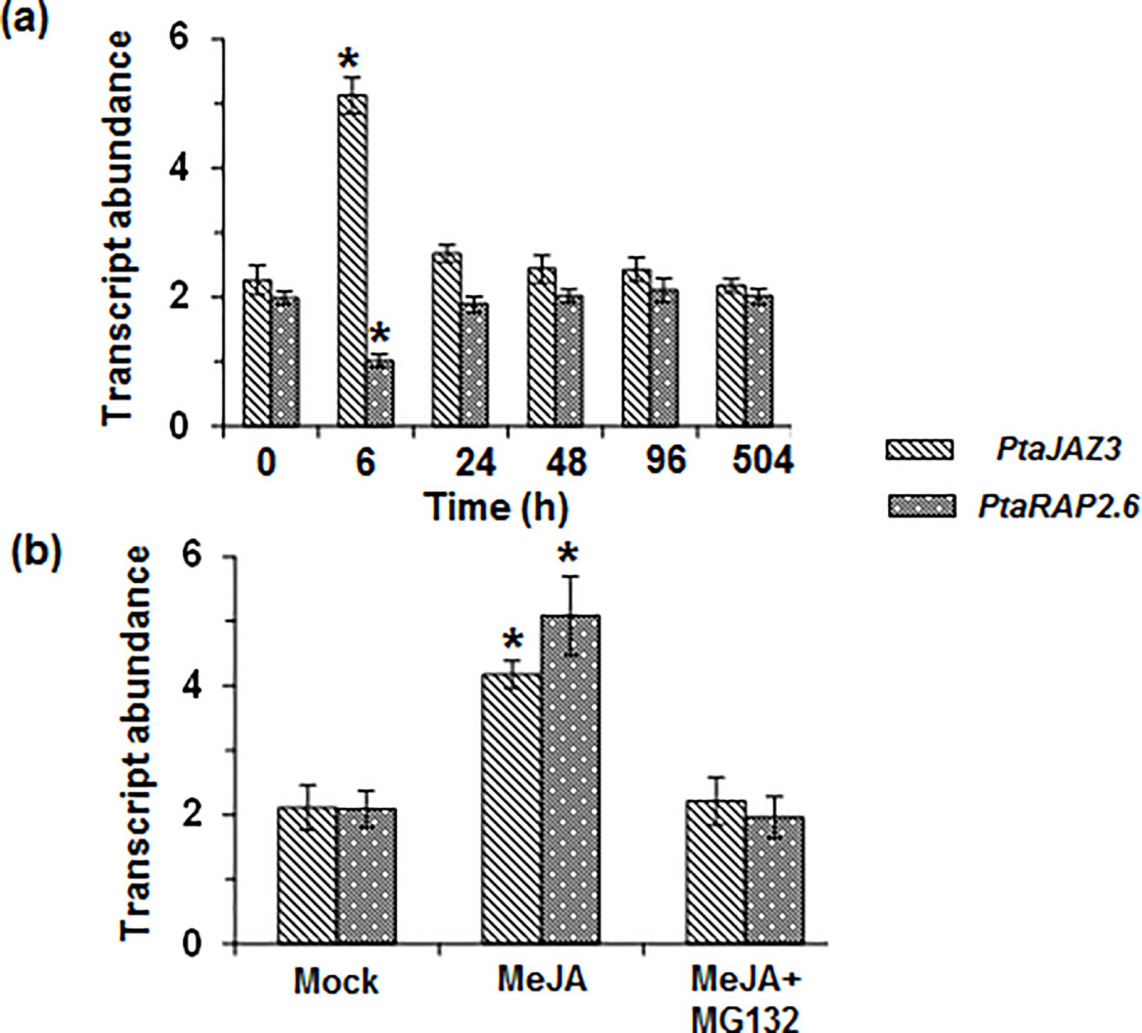
Drought stress and methyl jasmonate treatment affect *PtaJAZ3* and *PtaRAP2*.*6* expression. (a) Transcript levels of *PtaJAZ3* and *PtaRAP2*.*6* in response to PEG-induced drought stress. Poplar roots grown in control and PEG liquid media (see experimental procedures) were used for qRT-PCR analysis. Values are show as mean ± standard error of mean (SEM) (*n* = 2). Asterisks represent statistical significant levels of differences between control and PEG treatment (*P*< 0.05) calculated using Student’s *t* test. (b) *PtaJAZ3* and *PtaRAP2*.*6* are induced in response to methyl jasmonate (MeJA) treatment but the induction of both genes was abolished in presence of proteasome inhibitor, MG-132. Transcript levels were analyzed in poplar root samples treated with 0.2% DMSO (mock), 100 μM MeJA and 100 μM MeJA+ 80 μM of MG-132 (see experimental procedures). Values show as mean ± standard error of mean (SEM) (*n* = 2). Asterisks represent the treatments that are statistically different from the mock treatment (*P*< 0.05) calculated using Student’s *t* test.

Based on the microarray data, both PtaJAZ3 and PtaRAP2.6 show up-regulation at 6h (S1 Data). However, based on the qRT-PCR analysis, both *PtaJAZ3* and *PtaRAP2*.*6* responded early (6h), but in an opposite fashion to the drought stress treatment (PEG) (Fig 6A). *PtaJAZ3* expression significantly increased whereas *PtaRAP2*.*6* expression significantly decreased in response to the PEG treatment at 6 h (Fig 6A).The response to PEG, for both genes, was short-lived as their expression reverted to pre-treatment levels as soon as 24h after the initiation of the treatment both for qRT-PCR (Fig 6A) and microarray analysis (S1 Data). Because PtaJAZ3 is a homolog of the *Arabidopsis* JAZ3, which has been implicated in jasmonate signaling [50, 51], we studied if the expression of the two genes was affected by this hormone. Indeed, both *PtaJAZ3* and *PtaRAP2*.*6*transcript levels were highly induced by methyl jasmonate (Me-JA) treatment (Fig 6B). JAZ3 levels have been shown to be controlled by ubiquitin mediated proteasome degradation resulting in Me-JA-mediated induction of *JAZ3* transcript level[50]. We, therefore, investigated if the increase in transcript levels of the two genes were dependent on proteasome degradation and affected by the treatment with a strong proteasome inhibitor (i.e., MG-132). We found that treatment with MG-132 essentially eliminated the Me-JA induction in the expression of both genes (Fig 6B).

### *PtaJAZ3* transgenic overexpression affects expression of *PtaRAP2*.*6*

**Fig 7:**
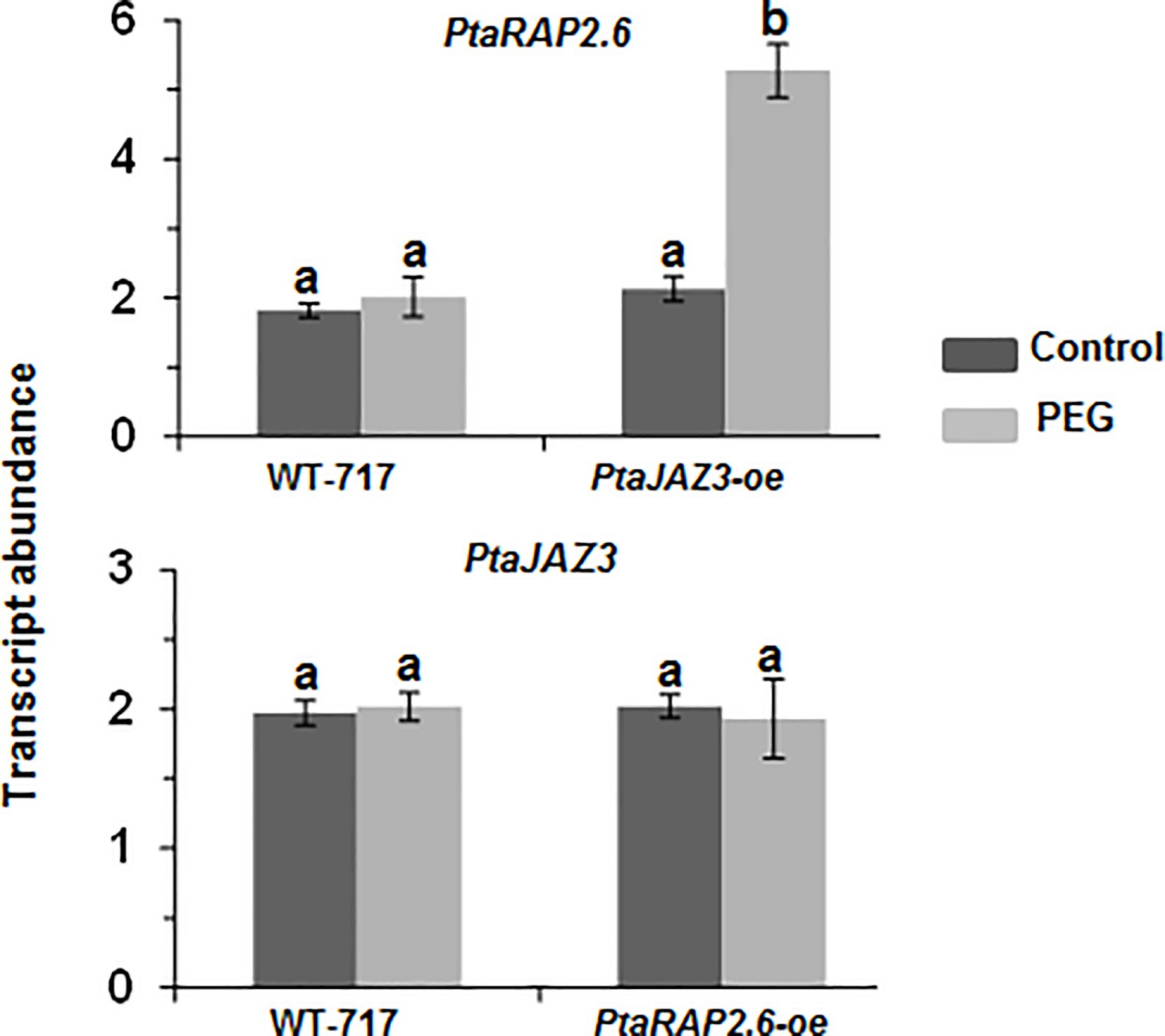
*PtaRAP2*.*6* expression was increased in response to PEG treatment in *PtaJAZ3* overexpressing lines. Expression of *PtaJAZ3* (top) and *PtaRAP2*.*6* (bottom) was analyzed in root samples of WT-717 and *PtaRAP2*.*6-*overexpression (*oe*) and *PtaJAZ3-oe* transgenic lines, respectively, growing in control and PEG liquid media. Two independent lines (4 replicates per line) were measured for calculating the value of each transgenic modification that is shown as mean ± standard error of mean (SEM) (*n* = 2). Different letters represent those means that are statistically different (*P*< 0.05) as determined by a one-way ANOVA followed by Tukey’s multiple range tests.

Because of the strong indication that the two superhub genes might be part of a common regulatory network, we studied if they affected each other’s expression when transgenically modified. *PtaJAZ3* was not affected in *PtaRAP2*.*6-oe* lines but the expression of *PtaRAP2*.*6* was significantly increased in *PtaJAZ3-oe* lines and this again occurred only in response to PEG treatment (Fig 7), indicating there is a regulatory interaction between the two superhubs.

## Discussion

PEG-induced drought stress causes a significant root growth inhibition in poplar[24]. We were interested in identifying key regulators that, when transgenically modified, can overcome this inhibition. Earlier studies have applied global transcriptomic approaches to study drought stress in root and foliar tissues of poplar [25, 31]. However, in contrast to these previous studies, which characterized the stress response in one time point, here we followed the transcriptomic changes during the temporal progression of the response. As we have shown previously with low nitrogen [38, 40] following the response in time allows inference of the hierarchical regulatory relationships among the differentially expressed genes. Indeed our analysis identified a hierarchical structure centered on 9 genes, which we called superhubs, because they are primarily connected to hub genes, which are then ultimately connected to approximately 3,000 DEGs (more than half of the differentially regulated transcriptome). We were successful in developing transgenic plants (overexpressing) for two of these superhubs, *PtaJAZ3* and *PtaRAP2*.*6*. Overexpression of both *PtaJAZ3* (*PtaJAZ3-oe*) and *PtaRAP2*.*6*(*PtaRAP2*.*6-oe*) overcame root growth inhibition in response to PEG-induced drought stress. Both *PtaJAZ3* and *PtaRAP2*.*6* were further connected to four hub genes, respectively. Except for *PtaARFA1F*, all other hub genes connected to *PtaJAZ3* displayed an increase in transcript levels in *PtaJAZ3-overexpression* (*oe*) lines and mostly in response to PEG treatment only. Similarly, hub genes connected to *PtaRAP2*.*6* also displayed an increase in transcript levels in *PtaRAP2*.*6-oe* lines only under PEG-induced drought conditions. Therefore, overexpression of both genes caused increased transcript levels of the putative downstream hub genes under PEG treatment conditions. However, studies in *Arabidopsis* show that the two genes encode proteins with different transcription regulatory activities. The putative *Arabidopsis* ortholog of *PtaJAZ3* has been shown to act as a transcriptional repressor [50, 52] whereas, RAP2.6 belong to AP2/ERF family of transcription factors and has been shown to act as a transcriptional activator in *Arabidopsis* [53]. This suggests that the effect of *PtaJAZ3* to its putative hub genes was indirect and was likely mediated by additional regulatory levels, likely following a double de-repression mode (repress of repressor). Since PtaRAP2.6 is an activator, we could not rule out the possibility that some of the hub genes were direct targets but additional study is needed to validate this assumption.

JAZ proteins including JAZ3 are part of the jasmonate signal transduction pathway and Transcript levels of *JAZ* genes have been shown to be induced in response to jasmonate treatment [54–56]. Similarly, we also observed an increase in *PtaJAZ3* transcript level in response to jasmonate treatment. Jasmonate signaling has been shown to be involved in abiotic stress responses [57, 58] as well as root growth [59, 60]. Recently, it has been demonstrated that salt stress inhibited root growth in *Arabidopsis* and this effect was also associated with activation of jasmonate signaling pathway and up-regulation of *JAZ* genes as a result of degradation of JAZ protein [61]. Similarly, we also observed an increase in *PtaJAZ3* expression as early as 6h after PEG treatment and, therefore, it is likely that up-regulation of *PtaJAZ3* gene was due to PtaJAZ3 protein degradation.

Though the effect of jasmonate treatment on root growth [61]inhibition and JAZ proteins degradation [50, 56] is known, not much is known regarding *JAZ* genes effect on root growth. Overexpression of *JAZ* genes in *Arabidopsis* displays no phenotype [56] except *JAZ10* whose overexpression resulted in strong jasmonate insensitivity [55]. We found that transgenic upregulation of *PtaJAZ3* had a strong positive effect on root development and this effect was specific to drought stress conditions. The exact mechanism of the *PtaJAZ3* effect is still unclear but it is likely that the observed *PtaJAZ3-oe* phenotype was mediated at least in part via *PtaRAP2*.*6*upregulation. This is suggested by the fact that *PtaJAZ3* overexpression led to an increase in transcript levels of *PtaRAP2*.*6* and *PtaRAP2*.*6* overexpression resulted in a similar positive effect on root development under drought stress.

In *Arabidopsis*, *RAP2*.*6*has been shown to be associated with both biotic [62] and abiotic stress (salt and drought)responses [53, 63]. The response of *RAP2*.*6* to stress was mediated at least in part by ABA signaling [53, 63]. However, recent evidence shows that RAP2.6 is also downstream of the jasmonate signaling [64] and jasmonate signaling has been implicated in mediating salt and osmotic stress, particularly with respect to root development [61]. Our data also indicate that *PtaRAP2*.*6* is likely a part of jasmonate signaling as it is inducible by methyl jasmonate treatment.

Though there is a significant amount of evidence about RAP2.6’srole in abiotic stress responses [53, 63], little is known regarding its role in root growth. In *Arabidopsis*, *RAP2*.*6* is ubiquitously expressed [53] and overexpression of the gene results in dwarf phenotype with increased salt and osmotic stress tolerance [63]. However, *RAP2*.*6* has a close paralog (*RAP2*.*6L*), which when overexpressed confers stress tolerance but no pleiotropic growth defects [63]. Thus, although the two paralogous genes share the function to confer stress tolerance, they have clearly diverged with respect to effects on overall growth and development. It is possible that similarly, the poplar *PtaRAP2*.*6* gene have evolved a very specific function in relation to root development. In support to this,*PtaRAP2*.*6* is almost exclusively expressed in roots though its *Arabidopsis* homologs show ubiquitous expression [53, 54, 63].

## Conclusions

In summary, our study has identified two new genes, *PtaJAZ3* and *PtaRAP2*.*6*, which, when overexpressed, increased root growth in poplar only under PEG-induced drought condition. The discovery of the two genes and the evidence we obtained could significantly advance our understanding of the underlying mechanisms. Further study and elucidation of the underlying mechanisms will help in developing new technologies for increasing drought tolerance in poplar and possibly other crops.

## Supporting information

S1 Fig Real time qRT-PCR validation of the expression changes in a subset of genes as measured by the microarray analysis. *PtaNRAMP1* (Potri.005G181100; Natural Resistance-Associated Macrophage Protein 1), *PtaB12D* (PtpAffx.216900.1.S1_s_at; Potri.017G098800; Barley aleurone and embryo protein), *PtaNAS4* (Potri.004G193400; Nicotianamine Synthase), *PtaMIPS* (Potri.005G078700; Myo-Inositol 1-Phosphate Synthase), *PtaXTR6* (Potri.006G170100; Xyloglucan Endotransglycosylase 6), *PtaTIP1;3* (Potri.009G027200; Tonoplast Intrinsic Protein 1;3), *PtaEFH* (PtpAffx.36054.1.S1_at; Potri.002G219000; EF-hand family protein), *PtaEXTL* (PtpAffx.9055.2.S1_s_at; Potri.010G072200; Extensin-like protein),*PtaACO1* (PtpAffx.206393.1.S1_s_at; Potri.006G151600; 1-Aminocyclopropane-1-Carboxylate Oxidase) and *PtaJAZ3*(PtpAffx.8326.2.A1_at; Potri.010G108200; Jasmonate-Zim-Domain protein 3).
The above values represent fold-change between control and PEG-treated poplar root samples at 6h and 24h after treatment. Values show mean ± SEM (*n* = 2).Asterisk represents significant difference from control (or DEG) for the given time-point (6h or 24h) and method (qRT-PCR or affymetrix) used. qRT-PCR was performed on the same root samples that were used for microarray analysis. Ubq was amplified as a normalization control.
(TIF)

S2 Fig Poplar root transcriptome in response to PEG stress is hierarchically structured around nine superhub genes. The gene regulatory network is constructed based on transcription profiling data of poplar roots grown under control and PEG conditions. Individual genes are represented as nodes whereas the edges/lines represent connections between the genes. The 9 superhub genes are represented as green square-shaped nodes, hubs as red hexagon whereas terminal genes are shown as pink circles.
(TIF)

S3 Fig Validation of the transgenic overexpression of *PtaJAZ3* and *PtaRAP2*.*6* in three independent transgenic lines. RNA was extracted from roots of plants grown in control media and transcript abundance was analyzed using qRT-PCR. Values show mean ± SEM (*n* = 3). Asterisks represent lines that are statistically different from WT-717 (*P*< 0.05) calculated using Student’s *t* test.
(TIF)

S4 Fig Native expression of *PtaJAZ3* and *PtaRAP2*.*6* in different tissues. Transcript abundance was analyzed in WT-717 tissues. Values show mean ± SEM (*n* = 3) and different letters represent means that are statistically different (*P*< 0.05) as determined by a one-way ANOVA followed by Tukey’s multiple range tests.
(TIF)

S1 Table Primers used for the qRT-PCR gene expression analysis. (PDF)

S2 Table Primers used for cloning of the nine superhub genes. (PDF)

S3 Table Significantly enriched GO-term associated with the genes terminally connected to hubs associated with *PtaJAZ3* and *PtaRAP2*.*6* as shown in Fig 4. The number of genes connected to each hub is shown in the parenthesis. The agriGO tool (http://bioinfo.cau.edu.cn/agriGO/) was used to perform the enrichment analysis using SEA (Singular Enrichment Analysis) coupled with available background data of *Populus trichocarpa* genome data (V 3.0).Abbreviations for the eight hubs connected to *PtaJAZ3*and *PtaRAP2*.*6*are as follows: 1. *PtaCYCB2;4* (Cycline B2;4, Potri.005G251400); 2. *PtaSAUR37*(Small Auxin Upregulated RNA 37, Potri.006G278100); 3. *PtaARFA1F* (ADP-Ribosylation Factor A1F, Potri.008G100000); 4.*PtaGH16* (Glycosyl Hydrolases family 16, Potri.006G071200); 5. *PtaGRF* (Gibberellin-Regulated Family protein, Potri.014G020100); 6.*PtaEBF1* (EIN3-Binding F-box protein1, Potri.006G068500); 7. *PtaCDC2* (Cell Division Control 2, Potri.004G133500) and8. *PtaXTH23* (Xyloglucan Endotransglucosylase/Hydrolase 23, Potri.018G095100).
(PDF)

S1 Data Differentially expressed genes (DEGs) associated with poplar root transcriptome in response to PEG treatment. Rank product (RP) was used for identifying DEGs (see experimental procedures). Only genes with corrected p-values less than 0.05 (calculated using Benjamini and Hochberg False Discovery Rate) were selected as DEGs (highlighted yellow). Fold change represents the expression fold change between control and PEG treatment.
(XLSX)

S2 Data Significantly enriched (*P ≤* 0.05) GO terms associated with all DEGs. The agriGO tool (http://bioinfo.cau.edu.cn/agriGO/) was used to perform the enrichment analysis using SEA (Singular Enrichment Analysis) coupled with available background data of *Populus trichocarpa* genome data (V 3.0).
(XLSX)

S3 Data Genes associated with putative gene regulatory network constructed using transcription profiling data of poplar roots grown under control and PEG conditions. (XLSX)

S4 Data Details of the terminal genes associated with the hubs connected with *PtaJAZ3* and *PtaRAP2*.*6* as shown in Fig 4. (XLSX)
