# Supplementary figures and images for: Gene network analysis of poplar root transcriptome in response to drought stress identifies a PtaJAZ3PtaRAP2.6-centered hierarchical network
Source: PLoS One. 2018 Dec 12;13(12):e0208560. doi: 10.1371/journal.pone.0208560 (PMC6291141; doi:10.1371/journal.pone.0208560)

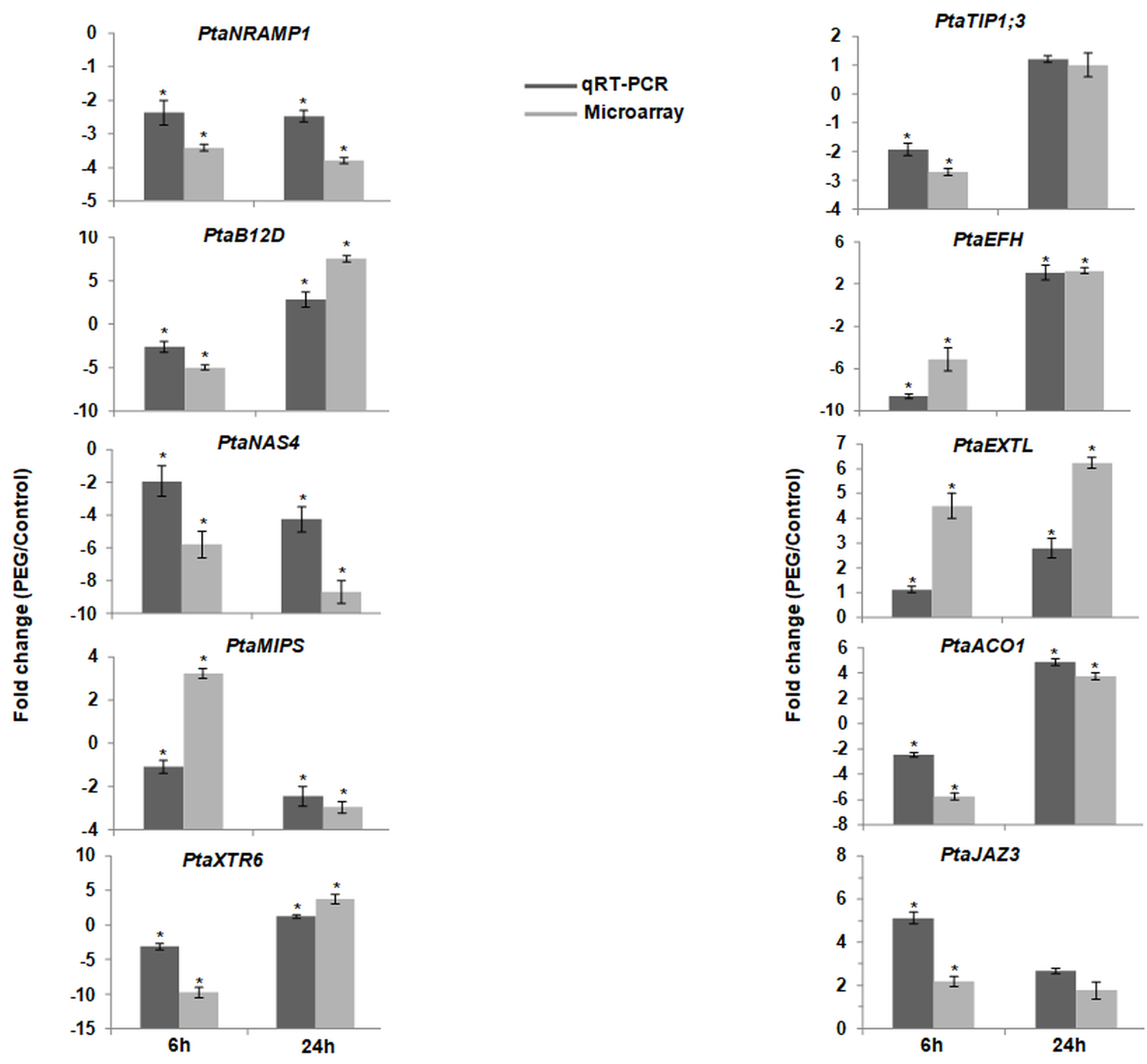

Supplement: S1 Fig — PtaNRAMP1 (Potri.005G181100; Natural Resistance-Associated Macrophage Protein 1), PtaB12D (PtpAffx.216900.1.S1_s_at; Potri.017G098800; Barley aleurone and embryo protein), PtaNAS4 (Potri.004G193400; Nicotianamine Synthase), PtaMIPS (Potri.005G078700; Myo-Inositol 1-Phosphate Synthase), PtaXTR6 (Potri.006G170100; Xyloglucan Endotransglycosylase 6), PtaTIP1;3 (Potri.009G027200; Tonoplast Intrinsic Protein 1;3), PtaEFH (PtpAffx.36054.1.S1_at; Potri.002G219000; EF-hand family protein), PtaEXTL (PtpAffx.9055.2.S1_s_at; Potri.010G072200; Extensin-like protein),PtaACO1 (PtpAffx.206393.1.S1_s_at; Potri.006G151600; 1-Aminocyclopropane-1-Carboxylate Oxidase) and PtaJAZ3(PtpAffx.8326.2.A1_at; Potri.010G108200; Jasmonate-Zim-Domain protein 3). The above values represent fold-change between control and PEG-treated poplar root samples at 6h and 24h after treatment. Values show mean ± SEM (n = 2).Asterisk represents significant difference from control (or DEG) for the given time-point (6h or 24h) and method (qRT-PCR or affymetrix) used. qRT-PCR was performed on the same root samples that were used for microarray analysis. Ubq was amplified as a normalization control. (TIF) [file pone.0208560.s001.tif]

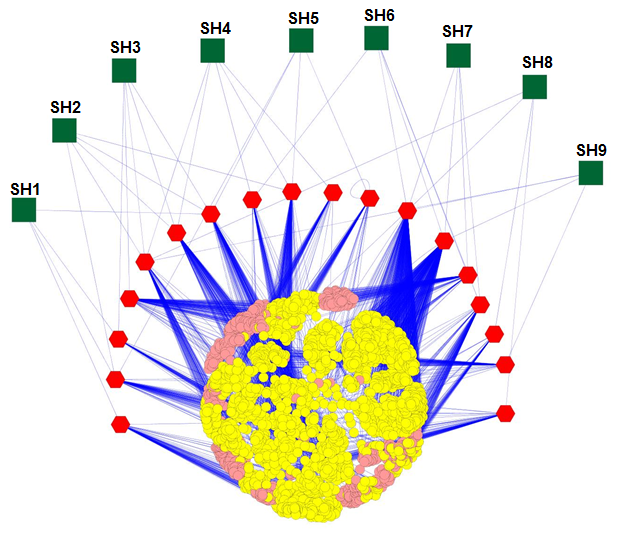

Supplement: S2 Fig — The gene regulatory network is constructed based on transcription profiling data of poplar roots grown under control and PEG conditions. Individual genes are represented as nodes whereas the edges/lines represent connections between the genes. The 9 superhub genes are represented as green square-shaped nodes, hubs as red hexagon whereas terminal genes are shown as pink circles. (TIF) [file pone.0208560.s002.tif]

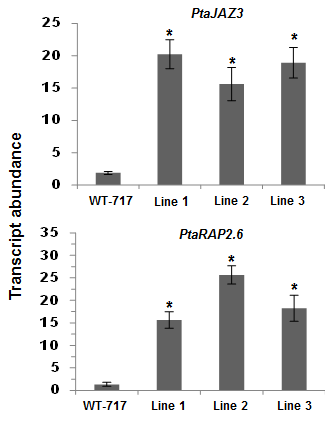

Supplement: S3 Fig — RNA was extracted from roots of plants grown in control media and transcript abundance was analyzed using qRT-PCR. Values show mean ± SEM (n = 3). Asterisks represent lines that are statistically different from WT-717 (P< 0.05) calculated using Student’s t test. (TIF) [file pone.0208560.s003.tif]

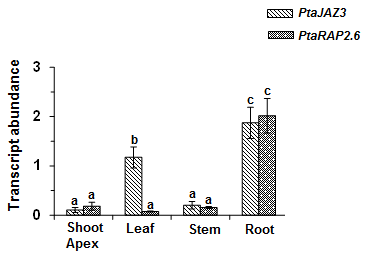

Supplement: S4 Fig — Transcript abundance was analyzed in WT-717 tissues. Values show mean ± SEM (n = 3) and different letters represent means that are statistically different (P< 0.05) as determined by a one-way ANOVA followed by Tukey’s multiple range tests. (TIF) [file pone.0208560.s004.tif]
