# Supplementary material for: Gene network analysis of poplar root transcriptome in response to drought stress identifies a PtaJAZ3PtaRAP2.6-centered hierarchical network
Source: PLoS One. 2018 Dec 12;13(12):e0208560. doi: 10.1371/journal.pone.0208560 (PMC6291141; doi:10.1371/journal.pone.0208560)
Supplement: S1 Table — (PDF) [file pone.0208560.s005.pdf]

S1 Table. Primers used for the qRT-PCR gene expression analysis.

Gene name	Poplar Gene ID (v 3.0)	Primer sequence
PtaJAZ3	Potri.010G108 200	F;TAAGTGCAGACGCATGTGATCC<br>R;GCGACCGGAAACATCTGGATTTC
PtaRAP2.6	Potri.001G067 600	F;ACCGACAATAACGTCCCAACGG<br>R;TGACGACGGATTGATGGAGAGC
PtaNRAMP1	Potri.005G181 100	F;TGCACTGGTTGTAGCACTCTGC<br>R;ACAGCTCTAGCTTCCTCACACC
PtaNAS4	Potri.004G193 400	F;ACTGCCTTGCCTGAAGAAGTGC<br>R;CCTCGGCATGGCCACATAAATTG
PtaXTR6	Potri.006G170 100	F;TCTTGGTCAAGAGTGCTACCGAAG<br>R;GCTTGTGGAAGATCCCAGTGAAAC
PtaUGT71B2	Potri.016G014 500	F;CAATGGCAATGGTCTGAGCC<br>R;ATCTCATCGTGGTATGGCCC
PtaMIPS	Potri.005G078 700	F;GTGCGTTTGTGTCGCATTGTGG<br>R;GCCATGTTACAACAGGCACACC
PtaTIP1;3	Potri.009G027 200	F;CCTCACAAAGGCTCCTCTTGTTCC<br>R;TTGCACGTTGCTTTGACAGTGC
PtaCCR	Potri.001G045 000	F;ACTTCCATGCCAAGGCCAAAC<br>R;AATTCTTGGGATCAGCTGGGTTTC
PtaB12D	Potri.017G098 800	F;GCGTTGGATGAAACCAGAGGTC<br>R;CAAACTTGTTACACAGGTCATCGC
PtaEFH	Potri.017G086 200	F;GGCTGTGGAGTGGGAATCTG<br>R;TTTGCTACATTCAGCTAAACTTGGA
PtaC4H	Potri.018G146 100	F;TTGGCATCGCCCAACATCCAAC<br>R;TTTGGCGAAGATTCTCCGAGAGG
PtaEXTL	Potri.010G072 200	F;CCCTGATTGCTTAGGGACACCATC<br>R;CGGCCACATTAGCATCGTTGAC
PtaEXPB1	Potri.003G083 200	F;ACTTTCATGGCCTTTGGTGGTG<br>R;TTTGATTGCTTGCCACCTGGAC
PtaCYCB2;4	Potri.005G251 400	F;TGGTTACGCAGCAAAGAACG<br>R;GGACAACCAATGAGAGACGGT
PtaSAUR37	Potri.006G278 100	F;ATCAAGAACCACTGCAGCTTCTAC<br>R;AGGAATCCTTAAGACACCTGCC
PtaARF1AF	Potri.008G100 000	F;TGGAGTGTCCGTCAAGTCAC<br>R;GGGTTTTATCTCCGGCACCT
PtaGH16	Potri.006G071	F;CTGGAAACTCTGCCGGAACT

	200	R;GGTGGGGTCGAACCAAAGAT
PtaGRF	Potri.014G020100	F;ATACCCTGTAGTATACCCAAGCTC<br>R;GTCACAGAATCCCGAGGCAG
PtaEBF1	Potri.006G068500	F;CCAATGTGGATGTATACAGCC<br>R; GCAACAGCAACCCTGTAGCTG
PtaCDC2	Potri.004G133500	F;TGGAAACTCCCACCTGAGAC<br>R;AAGCTTCAGCGCGTTGGTA
PtaXTH23	Potri.018G095100	F;CCCAACTGCTGATTTCCACA<br>R;GGCCCAATCTGTCTTGACCA
PtaUBQ	Potri.002G224700	F; AGAGTGTGAGAGAGAGAAGAG<br>R; CGACGACCATCAAACAAGAAG
