# Supplementary material for: Gene network analysis of poplar root transcriptome in response to drought stress identifies a PtaJAZ3PtaRAP2.6-centered hierarchical network
Source: PLoS One. 2018 Dec 12;13(12):e0208560. doi: 10.1371/journal.pone.0208560 (PMC6291141; doi:10.1371/journal.pone.0208560)
Supplement: S2 Table — (PDF) [file pone.0208560.s006.pdf]

S2 Table. Primers used for cloning of the nine superhub genes.

Superhub	Poplar Gene ID (v 3.0)	Primer name and sequence (5'→3')
Zinc Finger CCCH Domain Containing66/PtaCCCH66	Potri.001G252600	fPEG1;GGGACAAGTTTGTACAAAAAAGCAGGCTATGTGCAGTGGTAC rPEG1;GGGGACCACTTTGTACAAGAAAGCTGGGTTTCTAAGCCACTAATGG
High mobility group (HMG1/2) family/PtaHMG	Potri.003G138000	fPEG2;GGGACAAGTTTGTACAAAAAAGCAGGCTATGGCAGATACCGCT rPEG2;GGGGACCACTTTGTACAAGAAAGCTGGGTTTCTACTGCTGTTGTTTGTC
NUCLEAR RECEPTOR CO-REPRESSOR RELATED 1/PtaNCOR1	Potri.010G220000	fPEG3;GGGACAAGTTTGTACAAAAAAGCAGGCTATGCCGCCAGAGCCATTGC rPEG3;GGGGACCACTTTGTACAAGAAAGCTGGGTTCTACCTGCCTATGTCC
Myb-like transcriptional regulator family /PtaMYBL	Potri.016G001100	fPEG5;GGGGACAAGTTTGTACAAAAAAGCAGGCTATGGATCTGGATTGTGAAGC rPEG5;GGGGACCACTTTGTACAAGAAAGCTGGGTCTAAAGGTCACTGCCTAAGA
JASMONATE-ZIM-DOMAIN 3/PtaJAZ3	Potri.010G108200	fPEG4;GGGACAAGTTTGTACAAAAAAGCAGGCTATGGAAAGAGATTTTTTGGG rPEG4;GGGGACCACTTTGTACAAGAAAGCTGGGTTTAGTAGGGTTCGGGGTTGG
PtabZIP61	Potri.019G091900	fPEG6;GGGGACAAGTTTGTACAAAAAAGCAGGCTATGAGGAGAGGTCAGAAGTG rPEG6;GGGGACCACTTTGTACAAGAAAGCTGGGTTTATTGGGGTTTGCCATCAT
RELATED TO AP2.6/PtaRAP2.6	Potri.001G067600	fPEG7;GGGACAAGTTTGTACAAAAAAGCAGGCTATGTCTTCTATGGTTTCAGC rPEG7;GGGGACCACTTTGTACAAGAAAGCTGGGTTTACTCGCTAGGATTACTGG
zinc finger (C3HC4-type) RING finger family	Potri.008G134900	fPEG8;GGGGACAAGTTTGTACAAAAAAGCAGGCTATGGAGAAGGACATGGAAGG rPEG8;GGGGACCACTTTGTACAAGAAAGCTGGGTTCAATATGCAGGCCAGTAGT

/PtaC3HC4L		
NAC Domain Containing 028/PtaNAC028	Potri.017G086200	fPEG9;GGGGACAAGTTTGTACAAAAAAGCAGGCTATGTCATCCGAAAATGATTC<br>rPEG9;GGGGACCACTTTGTACAAGAAAGCTGGGTTCATACCTGTTCATTTCCCT
