# Supplementary material for: Gene network analysis of poplar root transcriptome in response to drought stress identifies a PtaJAZ3PtaRAP2.6-centered hierarchical network
Source: PLoS One. 2018 Dec 12;13(12):e0208560. doi: 10.1371/journal.pone.0208560 (PMC6291141; doi:10.1371/journal.pone.0208560)
Supplement: S3 Table — The number of genes connected to each hub is shown in the parenthesis. The agriGO tool (http://bioinfo.cau.edu.cn/agriGO/) was used to perform the enrichment analysis using SEA (Singular Enrichment Analysis) coupled with available background data of Populus trichocarpa genome data (V 3.0).Abbreviations for the eight hubs connected to PtaJAZ3and PtaRAP2.6are as follows: 1. PtaCYCB2;4 (Cycline B2;4, Potri.005G251400); 2. PtaSAUR37(Small Auxin Upregulated RNA 37, Potri.006G278100); 3. PtaARFA1F (ADP-Ribosylation Factor A1F, Potri.008G100000); 4.PtaGH16 (Glycosyl Hydrolases family 16, Potri.006G071200); 5. PtaGRF (Gibberellin-Regulated Family protein, Potri.014G020100); 6.PtaEBF1 (EIN3-Binding F-box protein1, Potri.006G068500); 7. PtaCDC2 (Cell Division Control 2, Potri.004G133500) and8. PtaXTH23 (Xyloglucan Endotransglucosylase/Hydrolase 23, Potri.018G095100). (PDF) [file pone.0208560.s007.pdf]

**S3 Table. Significantly enriched GO-term associated with the genes terminally connected to hubs associated with *PtaJAZ3* and *PtaRAP2.6* as shown in Fig 4.**

| Hubs | GO Ontology | Corrected p-values |
|---|---|---|
| *PtaCYCB2;3* (54 Genes) | GO:0043687: post-translational protein modification | 0.017 |
| | GO:0060255: regulation of macromolecule metabolic process | 0.018 |
| | GO:0010468: regulation of gene expression | 0.021 |
| | GO:0006139: nucleobase, nucleoside, nucleotide and nucleic acid metabolic process | 0.021 |
| | GO:0044260: cellular macromolecule metabolic process | 0.042 |
| *PtaSAUR37* (94 Genes) | GO:0010467: gene expression | 0.00029 |
| | GO:0034645: cellular macromolecule biosynthetic process | 0.0005 |
| | GO:0051726: regulation of cell cycle | 0.002 |
| | GO:0051171: regulation of nitrogen compound metabolic process | 0.0005 |
| | GO:0019222: regulation of metabolic process | 0.0012 |
| | GO:0009059: macromolecule biosynthetic process | 0.0043 |
| *PtaARFA1F* (117 Genes) | GO:0080090: regulation of primary metabolic process | 0.0029 |
| | GO:0019222: regulation of metabolic process | 0.0029 |
| | GO:0048527: lateral root development | 0.029 |
| | GO:0045449: regulation of transcription | 0.029 |
| | GO:0031323: regulation of cellular metabolic process | 0.031 |
| | GO:0050789: regulation of biological process | 0.37 |
| *PtaGH16* (59 Genes) | GO:0008152: metabolic process | 0.025 |
| | GO:0019219: regulation of nucleobase, nucleoside, nucleotide and nucleic acid metabolic process | 0.025 |
| | GO:0010468: regulation of gene expression | 0.025 |
| | GO:0007049: cell cycle | 0.039 |
| | GO:0045449: regulation of transcription | 0.045 |
| *PtaGRF* (70 Genes) | GO:0080090: regulation of primary metabolic process | 0.029 |
| | GO:0019222: regulation of metabolic process | 0.029 |
| | GO:0031326: regulation of cellular biosynthetic process | 0.029 |
| | GO:0031323: regulation of cellular metabolic process | 0.029 |
| | GO:0044249: cellular biosynthetic process | 0.029 |
| | GO:0045449:regulation of transcription | 0.029 |
| *PtaEBF1* (49 Genes) | GO:0031326: regulation of cellular biosynthetic process | 6.20E-05 |
| | GO:0031323: regulation of cellular metabolic process | 6.20E-05 |
| | GO:0045449: regulation of transcription | 6.50E-05 |
| | GO:0010468: regulation of gene expression | 6.50E-05 |
| | GO:0019219: regulation of nucleobase, nucleoside, nucleotide and nucleic acid metabolic process | 6.50E-05 |
| | GO:0051171: regulation of nitrogen compound metabolic process | 0.00015 |
| *PtaCDC2* (270 Genes) | GO:0044249: cellular biosynthetic process | 0.00069 |
| | GO:0055114: oxidation reduction | 0.00078 |
| | GO:0043687: post-translational protein modification | 0.0046 |
| | GO:0009059: macromolecule biosynthetic process | 0.0046 |
| | GO:0006950: response to stress | 0.0046 |
| | GO:0009987: cellular process | 0.007 |
| | | 0.007 |

| | | |
|---|---|---|
| *PtaXTH23* (31 Genes) | GO:0031326: regulation of cellular biosynthetic process | 0.009 |
| | GO:0031323: regulation of cellular metabolic process | 0.017 |
| | GO:0006807: nitrogen compound metabolic process | 0.017 |
| | GO:0045449: regulation of transcription | 0.027 |
| | GO:0006139: nucleobase, nucleoside, nucleotide and nucleic acid metabolic process | |

The number of genes connected to each hub is shown in the parenthesis. The agriGO tool (http://bioinfo.cau.edu.cn/agriGO/) was used to perform the enrichment analysis using SEA (Singular Enrichment Analysis) coupled with available background data of *Populus trichocarpa* genome data (V 3.0). Abbreviations for the eight hubs connected to *PtaJAZ3* and *PtaRAP2.6* are as follows: 1. *PtaCYCB2;4* (Cycline B2;4, Potri.005G251400); 2. *PtaSAUR37* (Small Auxin Upregulated RNA 37, Potri.006G278100); 3. *PtaARFA1F* (ADP-Ribosylation Factor A1F, Potri.008G100000); 4. *PtaGH16* (Glycosyl Hydrolases family 16, Potri.006G071200); 5. *PtaGRF* (Gibberellin-Regulated Family protein, Potri.014G020100); 6. *PtaEBF1* (EIN3-Binding F-box protein1, Potri.006G068500); 7. *PtaCDC2* (Cell Division Control 2, Potri.004G133500) and 8. *PtaXTH23* (Xyloglucan Endotransglucosylase/Hydrolase 23, Potri.018G095100).
